# Driving the Active Site Incorporation in Zeolitic Materials via the Organic Structure‐Directing Agent Through Development of H‐Bonds with Hydroxyl Groups

**DOI:** 10.1002/chem.202200702

**Published:** 2022-06-08

**Authors:** Beatriz Bernardo‐Maestro, Jian Li, Joaquín Pérez‐Pariente, Fernando López‐Arbeloa, Luis Gómez‐Hortigüela

**Affiliations:** ^1^ Molecular Sieves Group Instituto de Catálisis y Petroleoquímica, ICP-CSIC C/ Marie Curie 2 28049 . Madrid Spain; ^2^ Berzelii Center EXSELENT on Porous Materials Department of Materials and Environmental Chemistry Stockholm University Stockholm 10691 Sweden; ^3^ Departamento de Química Física Universidad del País Vasco Apartado 644 48080 Bilbao Spain

**Keywords:** active site distribution, aluminophosphate, refinement, structure-directing agent, zeolite

## Abstract

(1*S*,2*S*)‐N‐methyl‐pseudoephedrine (MPS) was used as organic structure‐directing agent (OSDA) for the synthesis of Mg‐doped nanoporous aluminophosphates. This molecule displays a particular conformational behavior, where the presence of H‐bond donor and acceptor groups provide a rigid conformational space with one asymmetric conformation preferentially occurring. MPS drives the crystallization of Mg‐containing AFI materials. Characterization of these materials shows that the OSDA incorporate as protonated species, arranged as head‐to‐tail monomers. Combination of three‐dimensional electron diffraction with high‐resolution synchrotron powder X‐ray diffraction allowed to locate both the Mg and the organic species. Interestingly, results showed that the spatial incorporation of Mg is driven by the hydroxyl groups of the organic cation through the development of H‐bonds with negatively‐charged MgO_4_ tetrahedra. This work demonstrates that H‐bond forming groups can be used to drive the spatial incorporation of low‐valent dopants within zeolitic frameworks, a highly desired aim in order to control their catalytic activity and selectivity.

## Graphical abstract

**(1*S*,2*S*)‐N‐methyl‐pseudoephedrine** used as organic structure‐directing agent for the synthesis of Mg‐doped aluminophosphates is able to control the spatial distribution of low‐valent dopants through the development of H‐bonds with MgO_4_ tetrahedra, providing a new strategy for controlling the active site spatial distribution in zeolite catalysts.

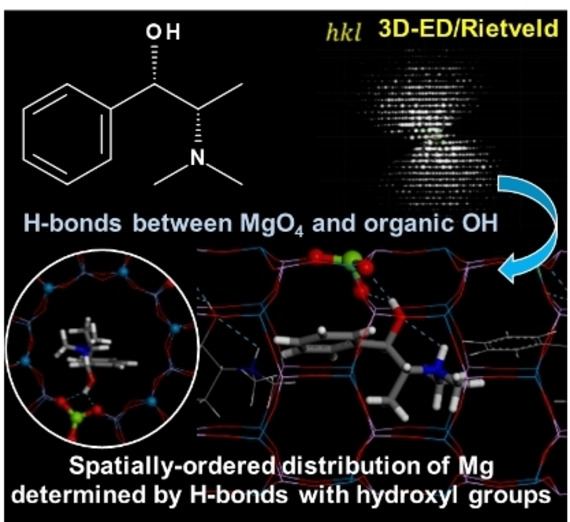

## Introduction

Zeolites are crystalline microporous materials composed of SiO_4_ and AlO_4_ tetrahedra that build 3‐dimensional frameworks with pores and cavities of molecular dimensions. In these materials, isomorphic substitution of Si^4+^ by low‐valent dopants like Al^3+^ or B^3+^ generates a negative charge in the framework that, after post‐synthesis treatments, can be compensated by a proton, generating Brønsted acid sites. Hence, the particular framework microporous structure of these materials, their hydrothermal stability and the potential incorporation of active sites confer them with optimal catalytic properties in a number of reactions, arising the characteristic shape‐selectivity.[^1^, ^2^, ^3^, ^4^] A related family of zeolite materials is provided by nanoporous aluminophosphates (AlPO_4_), where SiO_4_ tetrahedra are substituted by alternated AlO_4_ and PO_4_ tetrahedra.[^5^, ^6^] The particular chemistry of the so‐called AlPO_4_ networks enables a versatile incorporation of different catalytic active sites by replacement of either Al, P or both by other suitable elements, leading to efficient catalysts in different types of reactions.^[7]^

In a chemical transformation catalyzed by zeolites, the microporous structure of the particular zeolite framework determines the associated selectivity of the catalytic reaction for steric reasons, providing the shape‐selectivity to reactants, transition‐states or products as a function of their size/shape and the associated host‐guest fit with the pores and/or cavities of the framework.^[8]^ However, the particular location of the active sites embedded in the zeolite network can also influence the associated catalytic activity in complex framework structures where different crystallographic positions are available, giving the so‐called dopant‐sitting selectivity.[^9^, ^10^, ^11^, ^12^, ^13^] This is particularly true for zeolite frameworks where different cavities and/or porous elements are present, like was demonstrated for FER[^14^, ^15^, ^16^, ^17^] and MFI frameworks,[^18^, ^19^, ^20^, ^21^] among others. Therefore, attempting to control the spatial distribution of active sites in microporous frameworks represents a fundamental aim for controlling the activity and selectivity of zeolite catalysts.[^11^, ^22^, ^23^, ^24^] A particular case for these controlled spatial distributions of dopants involves the generation of chiral distributions of dopants embedded in zeolite frameworks that could potentially induce enantioselective properties on zeolite catalysts.^[25]^

The synthesis of zeolite materials usually involves the addition of organic cations that organize the inorganic units around, giving place to hybrid units from which crystallization of a particular framework takes place.[^26^, ^27^] These organic cations, which are referred as organic structure‐directing agents (OSDAs), remain occluded within the zeolite porous structure after the synthesis, filling the void space and providing additional stability to the hybrid system though host‐guest non‐bonding interactions. Thus, the versatility of the organic cations that can be used for the synthesis of zeolite materials prompts a strategy to attempt to control the incorporation of active sites in microporous networks.[^15^, ^24^]

The most usual isomorphic substitution in zeolitic materials involves the incorporation of a low‐valent dopant, such as trivalent dopants like Al^3+^ replacing Si^4+^ in zeolites, divalent dopants like Mg^2+^ replacing Al^3+^ in aluminophosphate networks, or even Si^4+^ replacing P^5+^ in silico‐aluminophosphate networks. These low‐valent isomorphic substitutions generate a negative net charge in the framework that is compensated by the positive charge of the OSDAs, most commonly being quaternary ammonium compounds, or by inorganic cations like Na^+^ or K^+^. Therefore, the development of strong electrostatic interactions between those negative network charges associated to the active sites and the positive charge associated to the quaternary ammonium groups or to inorganic guest cations has provided the main strategy to control the spatial distribution of dopants.[^15^, ^18^, ^19^] Indeed, we demonstrated this for the FER framework obtained by different OSDA cations, and we observed a strong influence over the catalytic properties.^[14]^ However, especially in zeolite synthesis that most frequently uses quaternary ammonium compounds, the influence of the positive charge associated to the N atom on the location of the negatively‐charged dopant is usually limited by the presence of four alkyl substituents which forces N to move away from the framework walls, restraining the potential ability to control the spatial incorporation of dopants through electrostatic interactions. For this reason, we propose to use alternative functional groups that could potentially strongly‐interact with low‐valent dopants. In this context, apart from electrostatic interactions between net opposite charges, a strong interaction would be provided by the development of H‐bonds. Therefore, we propose to use OSDAs with hydroxyl (−OH) moieties that, because of its chemical nature, always locate in terminal positions (hence with the possibility of siting close to the framework walls), in an attempt to control the location of low‐valent dopants through development of H‐bonds with the negative charge associated to the dopant.

**Scheme 1:**
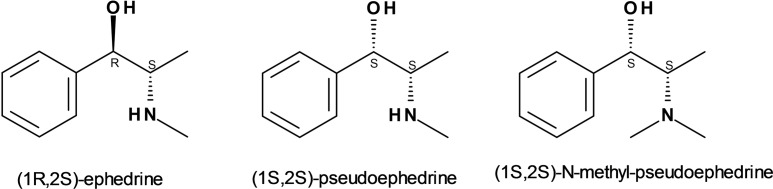
Molecular structure of ephedrine derivatives used as OSDAs in our group.

In line with the concept of controlling the spatial distribution of dopants, in a search for realizing chiral zeolitic materials, we have been recently working on a novel concept of chirality in zeolites, where we attempt to develop chiral distributions of dopants embedded within otherwise achiral zeolite frameworks.^[25]^ Based on this, we have been working for some time with alkaloids (1*R*,2*S*)‐ephedrine and its diastereoisomer (1*S*,2*S*)‐pseudoephedrine in the synthesis of nanoporous aluminophosphates (Scheme 1).[^28^, ^29^, ^30^, ^31^, ^32^, ^33^, ^34^] These molecules contain two potential H‐bond forming groups, the hydroxyl OH and the ammonium NH groups, which could potentially drive the incorporation of low‐valent dopants; however, so far we have not been able to demonstrate such a dopant‐siting control by the OSDA. In this work we present the use of a new OSDA derived of pseudoephedrine, (1*S*,2*S*)‐N‐methyl‐pseudoephedrine (MPS) (Scheme 1), for the synthesis of doped aluminophosphate frameworks, and we demonstrate that this OSDA is indeed able to control the location of the dopants.

## Results

### A) Synthesis and Physicochemical Characterization of MgAPO‐5/MPS

**Figure 1:**
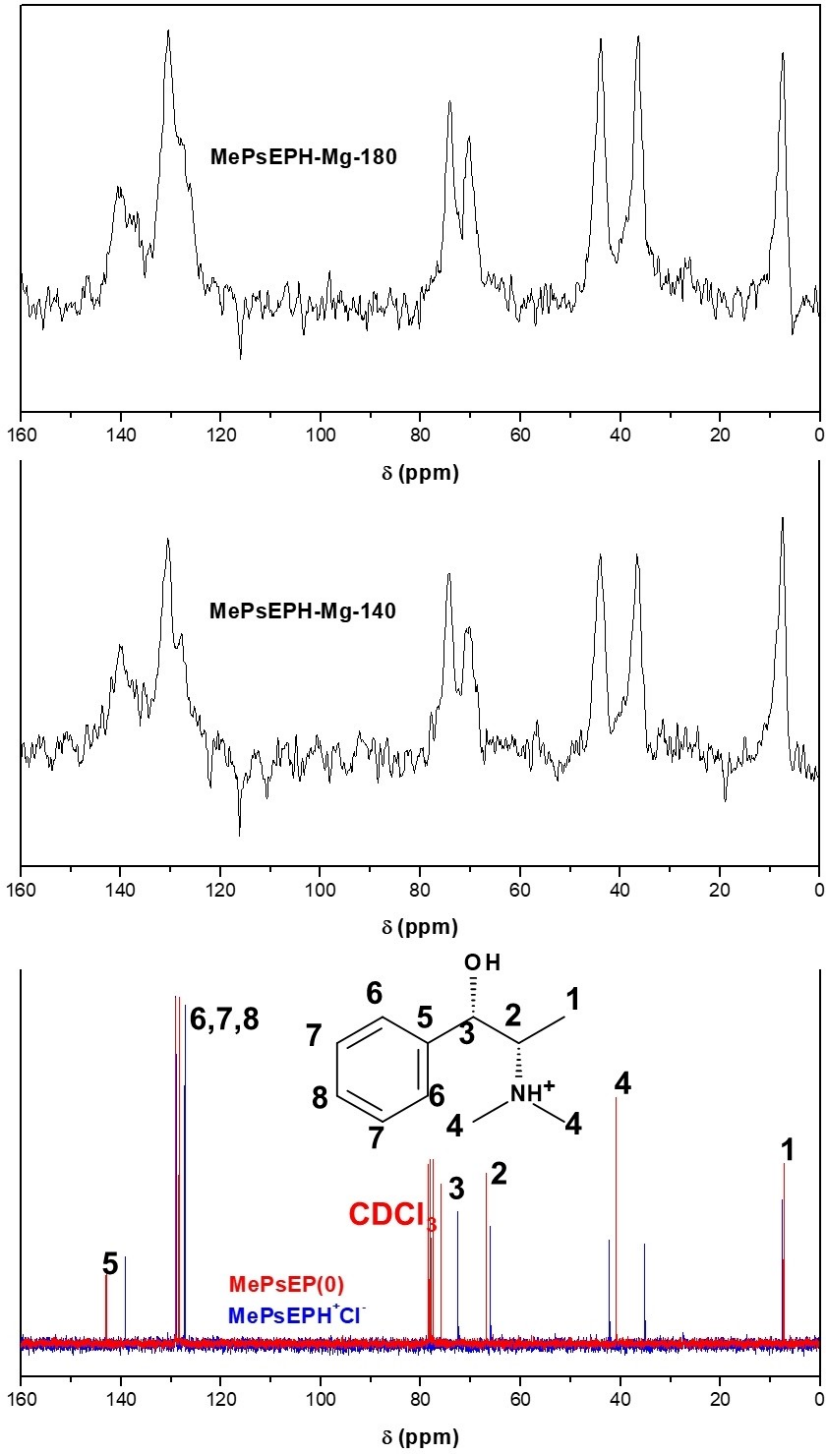
^13^C NMR of neutral (1*S*,2*S*)‐N‐methyl‐pseudoephedrine (bottom, in CDCl_3_, in red) and protonated (1*S*,2*S*)‐N‐methyl‐pseudoephedrinium chloride (in H_2_O, in blue), and of MgAPO‐5 obtained with (1*S*,2*S*)‐N‐methyl‐pseudoephedrine as OSDA at 140 °C (middle) and 180 °C (top) crystallization temperatures.

A systematic study of the structure‐directing effect of MPS showed that this molecule directs the crystallization of the AFI framework in the presence of Mg (MgAPO‐5); this structure comprises one‐dimensional non‐interconnected 12‐ring channels, with a diameter of 7.3 Å. Pure MgAPO‐5 materials were obtained from gels with molar composition 1MPS:1P_2_O_5_:0.22MgO:0.89Al_2_O_3_:50H_2_O, both at 140 and 180 °C crystallization temperatures for 24 h (Figure S1 in the Supporting Information). ^13^C CP MAS NMR was collected to identify the chemical nature of the organic component within the AFI solids (Figure 1). All the different resonances in the MgAPO‐5/MPS materials (both obtained at 140 or 180 °C, Figure 1 middle and top) were assigned to MPS by comparison with the ^13^C NMR spectra of the pure neutral molecule (1*S*,2*S*)‐N‐methyl‐pseudoephedrine (in CDCl_3_, Figure 1‐bottom, in red). However, we observed two signals at 36.6 and 44.1 ppm of similar intensity, which should correspond to the two chemically‐equivalent C4 atoms, whilst the corresponding resonance for the neutral molecule in solution gave one unique band at 40.9 ppm (red line). All the rest of resonances in the MgAPO‐5 materials were compatible with the molecular structure of MPS, which pointed to the molecule being integrally incorporated in the AFI framework, despite such difference. In order to unravel the origin for this difference, liquid ^13^C NMR spectra of MPS hydrochloride (i. e. of the protonated MPS molecule), in water was collected (Figure 1‐bottom in blue); in this case, the resonance of C4 gave correspondingly two different bands with similar intensity, in good agreement with the observations made in the solid materials. This clearly showed that the two methyl C4 atoms become not equivalent upon protonation, and hence that rotation around the CH‐NHMe_2_ bond is restricted, probably as a consequence of some particular conformation, as we will see below. Interestingly, ^13^C NMR of AFI materials obtained with (1*S*,2*S*)‐N,N‐dimethyl‐pseudoephedrinium derivative (with an additional methyl group, and hence no H‐bonded‐to‐N atom) (Figure S2 in the Supporting Information) showed a single resonance for the three corresponding methyl groups, evidencing that in this case the C−N bond is free to rotate, and that such singular NMR splitting is particular for MPS. These results also allowed us to unambiguously identify the charge state of MPS in MgAPO‐5 materials as protonated, which was required in order to compensate for the negative charge raised by the isomorphic substitution of Al by Mg.

Due to the harsh hydrothermal conditions that MPS molecules undergo during crystallization, and the importance of keeping their enantiomeric purity for building chiral zeolites, we analyzed their potential racemization during the hydrothermal treatment. As‐made MgAPO‐5 solids with occluded MPS molecules were dissolved with NaOH aqueous solution, and the organic material was extracted with diethyl‐ether. After roto‐evaporation, the oily organic product obtained was analyzed by ^1^H and ^13^C NMR, confirming that it was MPS (Figure S3 in the Supporting Information). Analysis of the optical rotation of the extracted MPS molecules gave a positive specific rotation of +53° (c=0.95 g/100 mL in methanol), in good agreement with the theoretical value of +46° for (1S,2S)‐enantiomers^[35]^ and the experimental value obtained from our synthesis (+45°), demonstrating the resistance of MPS to racemization.

Thermogravimetric analyses of MgAPO‐5 solids (Figure S4 in the Supporting Information) showed a very similar desorption profile for both samples (obtained at 140 and 180 °C). Neglecting high‐temperature dihydroxylation processes, the water and organic unit cell compositions, as deduced from TGA, were 3.6 H_2_O and 1.1 MPS per AFI unit cell for MPS−Mg‐140, and 2.3 H_2_O and 1.1 MPS per AFI unit cell for MPS−Mg‐180 (note that the OSDA contents could be slightly smaller because of the simultaneous high‐temperature dihydroxylation processes; measurement of CHN analysis was not possible for these samples due to their very strong acidity which prevented a complete combustion during analysis). Since ^13^C NMR indicates that MPS molecules are incorporated as protonated species, and because of charge‐balance reasons, and the absence of PO^−^ defects, as shown by ^31^P NMR (see below), the organic content should be similar to the Mg content; therefore, the incorporation of Mg in this system is limited by the amount of MPS molecules that can be efficiently hosted within the AFI framework, which turned to be ∼1 MPS/u.c., as evidenced by the structure‐solution study (see below, section D), in good agreement with the TGA results.

**Figure 2:**
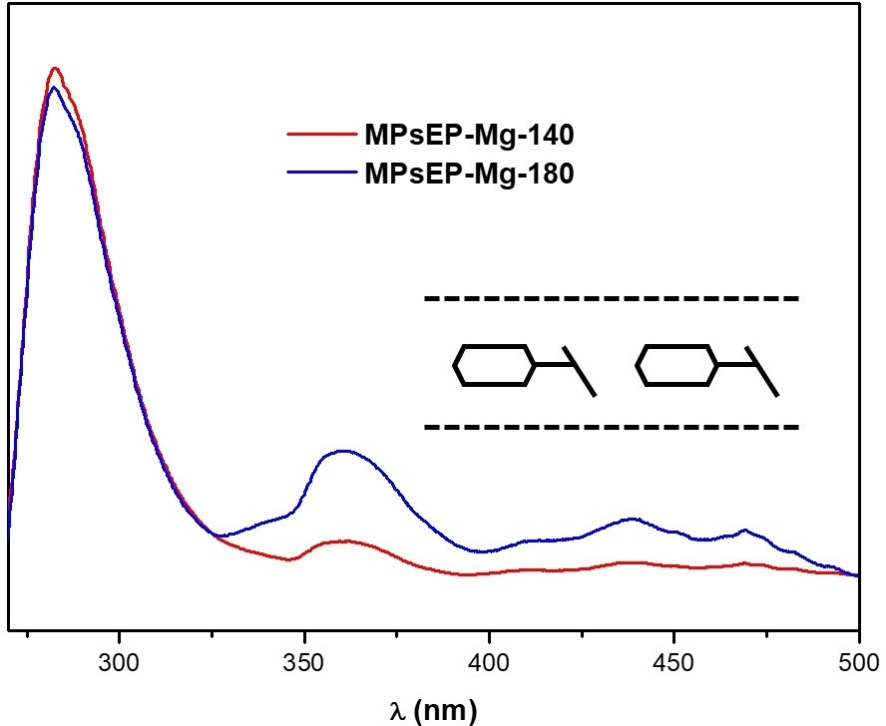
UV‐Vis Fluorescence spectra, after excitation at 256 nm, of MgAPO‐5/MPS materials obtained at 140 °C (red) or 180 °C (blue) crystallization temperatures. Inset: scheme of the MPS head‐to‐tail monomer configuration.

Due to the presence of an aromatic ring, MPS molecules could in principle pack within the AFI channels as dimers, stabilized through π‐π type interactions, as we have already observed for related (1*R*,2*S*)‐ephedrine and (1*S*,2*S*)‐pseudoephedrine.[^29^, ^32^] This can be easily monitored by UV‐Vis fluorescence spectroscopy, where a red‐shift of the fluorescence band of the phenyl ring is observed upon π‐π stacking, giving broad bands between 350 and 500 nm when dimers are occluded. In contrast, our MPS‐MgAPO‐5 materials show the main fluorescence band at 282 nm with a vibronic structure, which is unambiguously assigned to MPS monomers (Figure 2). Some additional bands with much lower intensity are observed in the dimer fluorescence region at ca. 360, 440 and 470 nm, which must correspond to some minor MPS molecules arranged as dimers; though clearly minority, these species are slightly more abundant in the sample obtained at high temperature, in good agreement with our previous observations in materials obtained with ephedrine and pseudoephedrine (these results are confirmed by UV‐Vis diffuse reflectance spectroscopy by the presence of shoulders between 300 and 400 nm for the sample obtained at 180 °C, see Figure S5 in the Supporting Information). We also note that a red‐shift of the fluorescence monomer band to 320 nm, keeping the vibronic structure, which we previously assigned to monomers developing co‐planar (rather than π‐π‐stacked) interactions,^[36]^ was not observed in this case. These results suggest that MPS protonated species pack within the AFI channels as monomers in a head‐to‐tail configuration (see inset in Figure 2), with the aromatic rings of consecutive molecules at opposite sides, preventing aromatic interactions.

SEM (Figure S6 in the Supporting Information) showed that MPS‐MgAPO‐5 materials crystallize as bouquet‐shaped symmetrical aggregates of smaller prismatic crystals. The crystal morphology is better defined in the sample obtained at 180 °C, where some hexagonal isolated prisms are also observed. In contrast, crystalline aggregates of the sample obtained at 140 °C are slightly larger.

**Figure 3:**
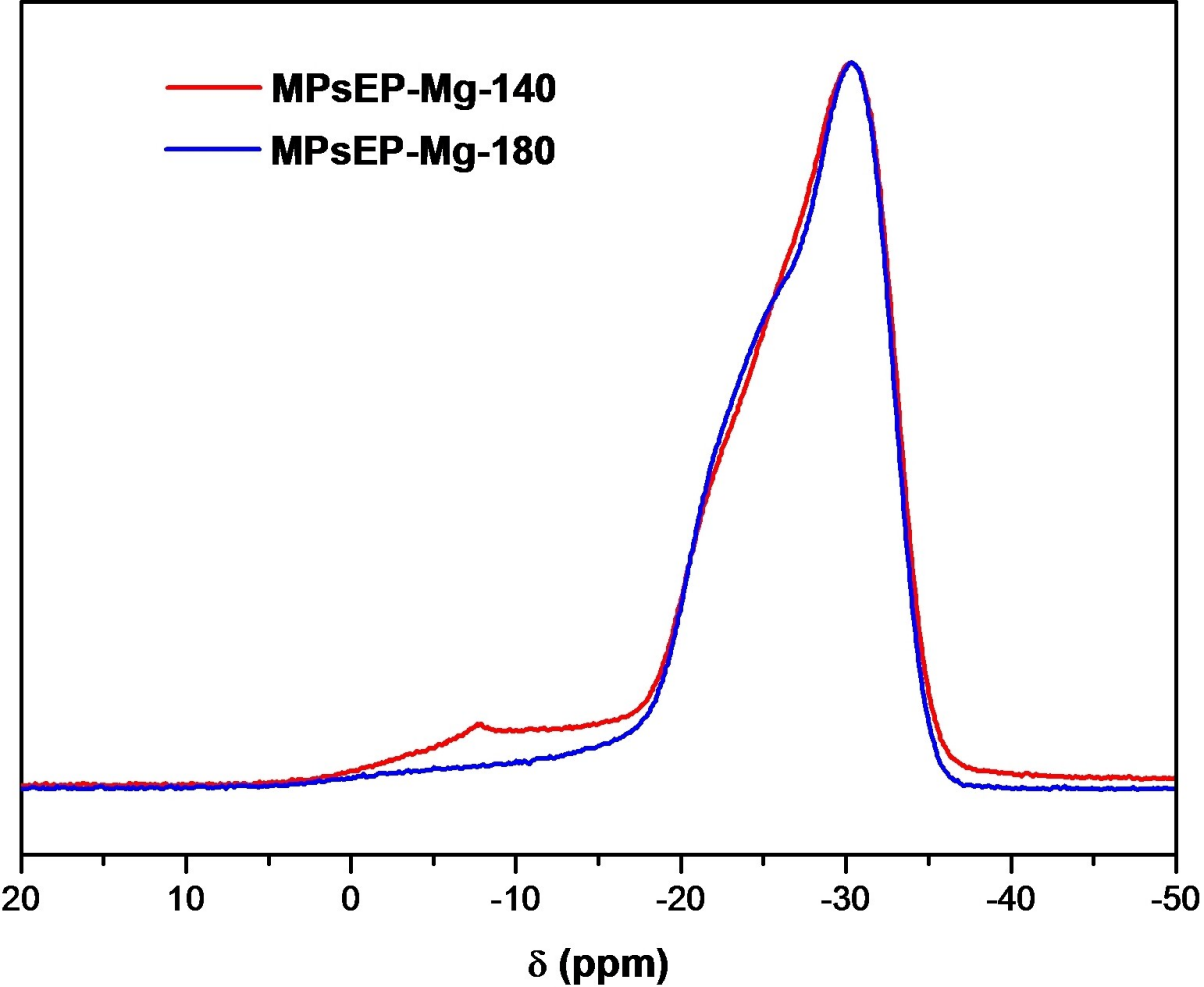
^31^P MAS NMR spectra of MgAPO‐5/MPS materials obtained at 140 °C (red) or 180 °C (blue) crystallization temperatures.

^31^P MAS NMR was used to analyze the incorporation of Mg in the AFI framework. Normal P (surrounded by 4 O(Al), P(4Al)) in the AFI network gives typically a signal at −30 ppm, which is shifted to higher chemical shifts when Mg is in the first coordination shell (to −24 ppm for P(1Mg,3Al) environments); P(2Mg,2Al) environments are less common because of the proximity of two charges, and give typically signals at −18 ppm. ^31^P MAS NMR spectra of our MPS‐MgAPO‐5 materials (Figure 3) display a signal at −30 ppm assigned to P(4Al). Interestingly, the profile for the P(1Mg3Al) environments of our materials show the contribution of a broad band between −26 and −20 ppm, which is clearly different from the typical P(1Mg,3Al) ^31^P bands observed for MgAPO‐5 materials, where a P(1Mg,3Al) resonance centered at −24 ppm is invariably observed (see Figure S7 in the Supporting Information for a comparison with typical MgAPO‐5 materials obtained with (1*R*,2*S*)‐ephedrine, (1*S*,2*S*)‐dimethylpseudoephedrinium or triethylamine). Such different P(1Mg) resonance might point to a particular configuration of the P−Mg units incorporated within the AFI network with MPS molecules, which is intrinsic to this host‐guest system. Collection of the ^31^P NMR spectrum under ^1^H‐CP conditions revealed a very similar profile, suggesting that those bands are not associated to P‐OH groups (Figure S8 in the Supporting Information). On the other hand, a minor band at ∼8 ppm observed for the sample prepared at 140 °C might be due to some amorphous/unreacted material, as evidenced by SEM (Figure S6‐top).

**Figure 4:**
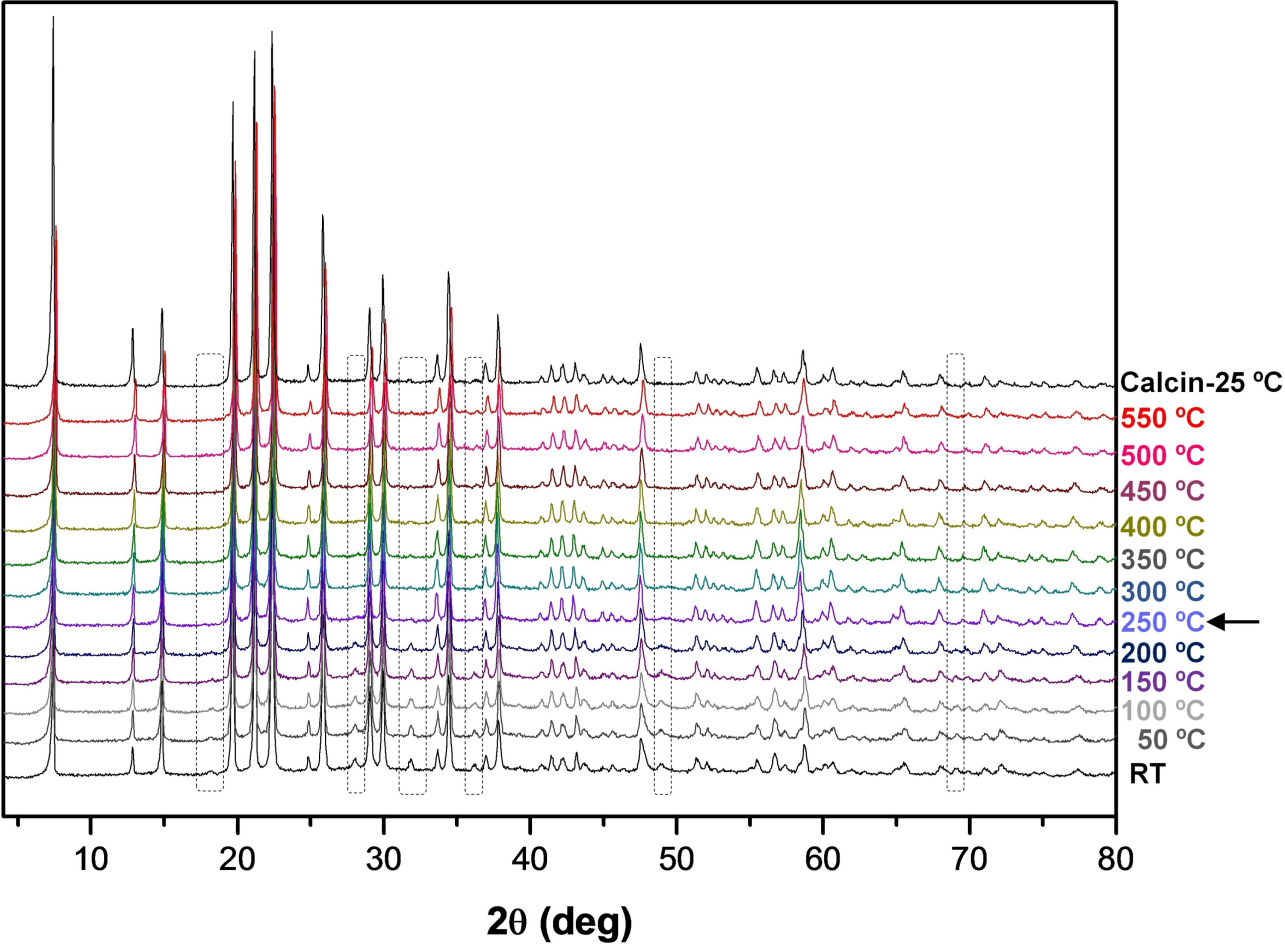
In‐situ XRD at increasing temperature of MgAPO‐5/MPS; peaks not assigned to conventional AFI framework that disappear upon calcination are highlighted within dashed rectangles.

We finally studied the elimination of the organic molecules by calcination. In‐situ XRD patterns at increasing temperatures were registered up to 550 °C, showing the resistance of the framework towards thermal degradation (Figure 4). In the XRD pattern of the as‐made samples, some additional peaks observed at 18.2, 28.1, 31.9, 36.2, 49.0, 55.0 and 69.2 2θ angles, which do not correspond to the conventional AFI framework, are invariably observed (dashed rectangles in Figure 4), and must be due to a particular symmetry associated to a singular ordering of the confined MPS molecules. Interestingly, these peaks progressively decrease their intensity from 150 °C, suggesting a thermally‐induced distortion of the organic configuration, which is then boosted at 250 °C when the organics start to desorb because of the generation of void volume. Such extra XRD peaks seem to be associated to the incorporation of the MPS cations as monomers (rather than dimers): we noted that in MgAPO‐5/MPS samples where a higher incorporation of dimers occurred (by increasing the MPS concentration in the gel and the crystallization temperature to 180 °C), the intensity of those peaks decreased (Figure S9 in the Supporting Information, red lines), suggesting a correspondence between occlusion of MPS as monomers and those extra diffraction peaks.

### B) Conformational Analysis of MPS

**Figure 5:**
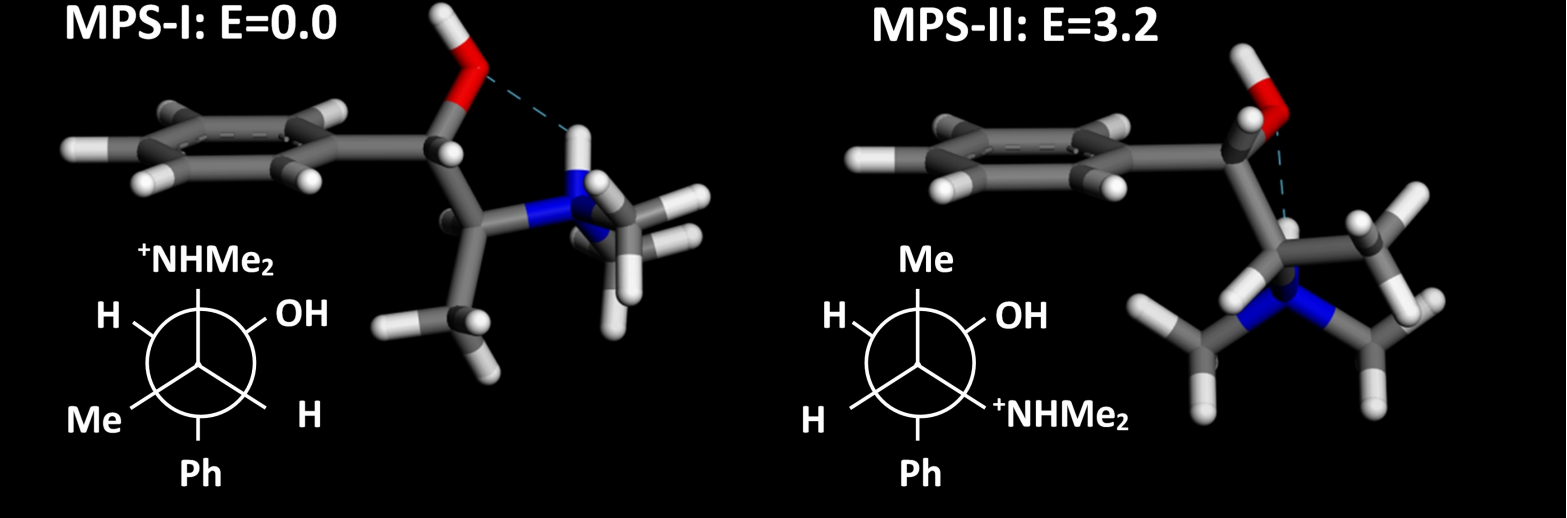
Two most stable conformers of protonated MPS, with the corresponding relative energies (calculated at the DFT/B3LYP level, in kcal/mol).

Due to the presence of H‐bond donor and acceptor groups within the molecule that will drive the conformational behavior of MPS, we performed a computational conformational study. Figure 5 shows the two most stable conformers of protonated MPS (with relative energies calculated at DFT/B3LYP level). There is one particular conformation that is clearly much more stable than the rest, MPS−I, that is stabilized by an intramolecular H‐bond interaction (Figure 5‐left). The next most stable conformer, MPS‐II, also displays an intramolecular H‐bond, but a strong steric hindrance (between bulky phenyl and ammonium groups) is apparent, as revealed by its rather lower stability (3.2 kcal/mol less stable) (Figure 5‐right). There are 5 additional conformers which are minima in the potential energy surface, but their stabilities are much lower (with relative energies ranging from 3.7 to 9.3 kcal/mol) due to the absence of intramolecular H‐bonds. Calculation of the conformational space with cvff forcefield or with DFT using plane waves and PBE functional (CASTEP) gave similar conformational energies (Table S1 in the Supporting Information).

Calculation of the ^13^C NMR chemical shifts (DFT/B3LYP) (Table S2 in the Supporting Information) did confirm the occurrence of different resonance bands for the methyl groups bonded to N (C4 in Figure 1) as a consequence of this particular conformation. Indeed, comparison of the calculated ^13^C chemical shifts of protonated MPS−I and MPS‐II with the experimental ones (in liquid solution) showed a stronger similarity of the latter with MPS−I predicted shifts (especially at C1 and C4 atoms, the ones mostly influenced by the particular conformation), which again suggests the occurrence of MPS in conformation I in solution.

### C) Ab initio Structure Determination of MgAPO‐5/MPS

**Figure 6:**
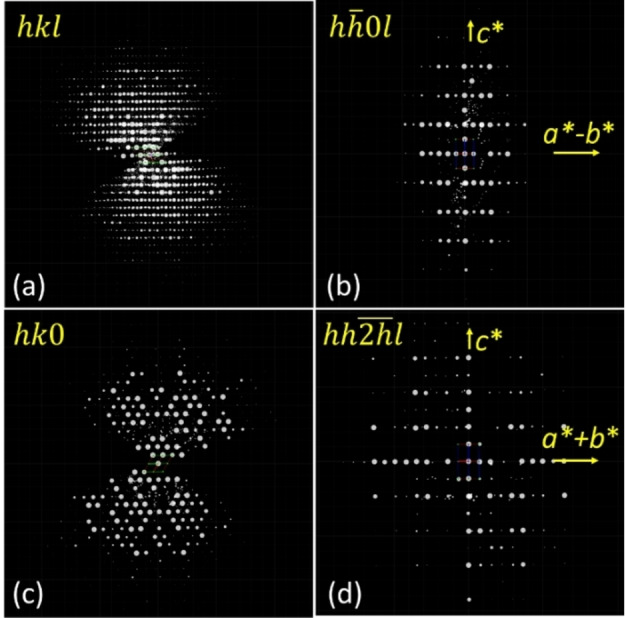
Reconstructed 3DED data of MgAPO‐5/MPS indexed with hexagonal symmetry: (a) overview, and selected planes in the reciprocal lattice corresponding to (b) $h\bar{h}0l$ , (c) hk0 and (d) $hh\bar{2h}l$ .

The XRD patterns of our MgAPO‐5/MPS materials, with those extra‐diffraction peaks, suggests a particular symmetry of these samples, probably due to a particular ordering of the OSDA cations inside the AFI channels. Hence, we were interested in trying to find the location of the organic cations and see if they have any influence on the incorporation of dopants, as we initially proposed. The structure of MgAPO‐5/MPS was determined by combining three‐dimensional electron diffraction (3DED) with high‐resolution synchrotron powder X‐ray diffraction (SPXRD). 4 datasets of 3DED were collected on isolated crystals with mean lattice parameters of a=13.7916(2) Å, c=8.3886(7) Å with hexagonal symmetry; the unit cell was similar to reference data in IZA database.^[37]^ However, after carefully analyzing the reflection conditions of 3DED, no special forbidden reflection was obtained, suggesting the possible space groups of *P*6(168#), *P*‐6 (174#), *P*622 (177#), *P*6mm (183#), *P*‐62m (189#), and *P*6/mmm (191#) (Figure 6). The 3DED datasets procession were then performed using XDS and scaled and merged based on unit cell consistency, correlation coefficients between the datasets, and resolution using XSCALE. Ab initio structure solution was performed by direct method using ShelxT; all Al, P and O atoms were located with a chiral space group of *P*6 directly. The chiral space group of *P*6 was further confirmed by Pawley profile‐fitting against SPXRD data. There are several weak peaks (e. g. 201, 4$\bar{2}1$ , 302) that could not be indexed using the normal symmetry of AFI with space group of *P*6/mcc, but perfectly matched with *P*6 (Figure S10 in the Supporting Information). The structure model of MgAPO‐5/MPS was then refined against the merged data using the lattice parameters obtained from the SPXRD data by the program Olex 2,^[38]^ and refined with atomic scattering factors for electrons, which resulted in reasonable average Al−O and P−O bond lengths. In the final stages of the refinement, the SWAT instruction was introduced to model the diffuse species in the channels (such as the OSDA). This instruction suppresses the contribution from the strong, low‐angle reflections where the contribution of extra‐framework species is strongest. This significantly reduced the R1 value, and the final refinement of the framework structure of MgAPO‐5/MPS converged with R_1_=0.1907, wR_2_=0.4513 and GoF=1.310.

### D) Location of Mg and OSDA and Rietveld refinement

**Figure 7:**
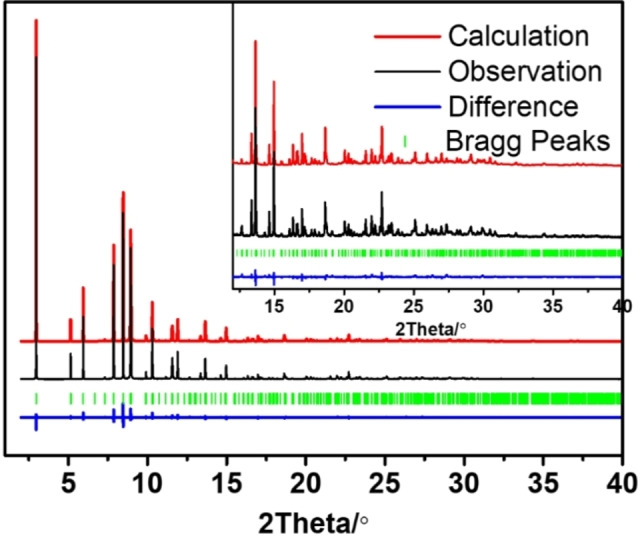
Rietveld refinement plot of MgAPO‐5/MPS. Black, red and blue curves show experimental, calculated, and difference values, respectively; vertical green lines indicate allowed reflections of MgAPO‐5/MPS in *P*6 symmetry. Deposition Number 2170196 for the structure of MgAPO‐5/MPS contains the supplementary crystallographic data for this paper. These data are provided free of charge by the joint Cambridge Crystallographic Data Centre and Fachinformationszentrum Karlsruhe Access Structures service.

**Figure 8:**
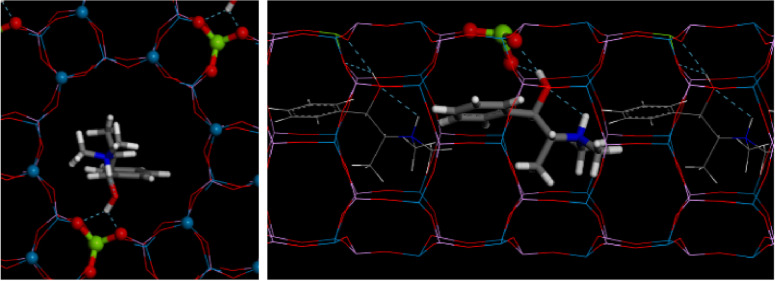
Final location obtained by Rietveld refinement of MgAPO‐5/MPS, showing only one of the six symmetry‐equivalent positions.

Understanding the host‐guest interaction of the chiral OSDA with the inorganic framework is crucial; this will indicate how the OSDA can direct the spatial incorporation of the dopants. However, the location of OSDAs and dopants like Mg represents a great challenge, even when high quality single‐crystal X‐ray diffraction data are available. The reasons are as follows. On the one hand, the OSDA consists of light scatterers and typically have low point symmetry, while the zeolite framework consists of heavier scatterers and have high symmetry, resulting in a lack of contrast to locate the OSDAs. Because of the difference in the symmetry between OSDAs and zeolite frameworks, the OSDA often appears to be disordered in the pores. On the other hand, the low content of dopants (Mg in this case) in the framework and similar X‐ray scattering power of Al and Mg makes the location of dopants very difficult. Combining the simulated annealing routine with Rietveld refinement has proven to be useful in locating organic guest molecules, where the algorithm uses the experimental diffraction pattern directly [40]. After the simulated annealing routine and Rietveld refinement, we managed to locate the MPS cation within the 12MR channels of the framework, with the hydroxyl group sitting close to the framework walls, indicating a strong H‐bond corresponding to O−H⋅⋅⋅O distances of 2.067(17) Å.^[39]^ The position of MPS matched well with the residual electron density inside the channel system. The Rietveld refinement resulted in a longer average Al−O distance of Al1 (1.66, 1.70, 1.74 and 1.78 Å) than Al2 (1.68, 1.69, 1.69 and 1.72 Å), which suggest that Mg should be located in Al1 as the Mg−O (theoretical bond length of ∼2.02 Å) is longer than for Al−O (∼1.72 Å). In addition, the Mg‐doped TO_4_ (Al1) sited close to the MPS hydroxyl group (Figure 8 and S11 in the Supporting Information), which developed a stable H‐bond with framework O's. With this model, the final refinement converged with R_wp_=0.1349, R_p_=0.1014 (Figure 7). The refinement details are shown in Tables S3‐S5, and a picture of the final refined location for MPS is shown in Figure 8 (showing only one of the 6 symmetry‐equivalent Mg⋅⋅⋅MPS pairs). Interestingly, the conformation adopted by MPS coincides with the most stable one found previously in the conformational analysis (MPS−I). We can clearly see the formation of an intramolecular H‐bond that determines the conformation adopted and intermolecular H‐bonds with the O atoms linked to the low‐valent dopant. We remark that all the extra‐peaks associated to the particular symmetry of MgAPO‐5/MPS disappear if the MPS cations are removed from the model, in good agreement with the observations made on the XRD patterns during the in situ XRD‐monitored calcination of the sample (Figure 4).

### E) DFT study of the Mg⋅⋅⋅MPS interaction

Our next step was to study by DFT methods the relative stability for the different possible locations of Mg in the MgAPO‐5/MPS system. Starting from the Rietveld results for the AFI/MPS location, we loaded one MPS cation (out of the 6 equivalent positions in the P6 symmetry), and Mg was located on one of the 12 Al positions in the AFI unit cell. The 12 different MgAPO‐5/MPS systems were then geometry‐optimized and the calculated relative energies as a function of the Mg position are shown in Table 1 (Figure S12 in the Supporting Information shows labelling for the different Al T positions; Al1‐Al6 are in the same 12‐ring where hydroxyl groups interact, and Al7‐Al12 are in the next 12‐ring of the unit cell). Results clearly show a higher stability for the incorporation of Mg in the position where an H‐bond with the hydroxyl group is developed, in good agreement with our refinement results (Figure 8). Indeed, OH(Mg)⋅⋅⋅Mg distances are shorter than N⋅⋅⋅Mg distances, suggesting that the H‐bond interaction (and not just the electrostatic interactions with the net charge of MPS or Van der Waals interactions) provides the main driving force for the particular Mg incorporation in this system, which is enabled by the singular molecular structure of MPS. Surprisingly, along our computational calculations we found another orientation for the MPS cations that was even slightly more stable (by 1.2 kcal/mol), where a double H‐bond interaction was developed between the framework O atoms surrounding Mg and both the hydroxyl and ammonium groups of the cation (Figure S13 in the Supporting Information). However, such configuration should give additional XRD peaks that have not been found in our study, showing that not always the most thermodynamically favored positions are reached, but kinetic factors during crystallization must impose the alternative orientation in our system, where the ammonium group sites in the middle of the channel.

**Table 1:** DFT calculated relative energy for different positions of Mg replacing Al in the AFI unit cell; position 1 is the one found by Rietveld, where an H‐bond is developed with the MPS hydroxyl group.

Mg	R.E. [kcal/mol u.c.]	Dist. Mg⋅⋅⋅O(MPS) [Å]
**1**	0.00	4.19
**2**	4.56	5.29
**3**	3.61	7.08
**4**	2.29	8.22
**5**	0.85	7.62
**6**	1.27	5.84
**7**	3.72	3.76
**8**	6.85	5.43
**9**	6.24	6.79
**10**	5.11	7.49
**11**	4.96	6.61
**12**	5.40	4.58

### F) Long‐range packing of MPS in Mg‐AFI

The P6 symmetry found previously and the location of MPS involves that consecutive molecules along the 12‐MR channel should be rotated by an angle of ±n ⋅ 60° (n=0, 1, 2, 3, 4 or 5: 0°, +60°, +120°, +180°, −120° or −60°). Hence, if a higher stability would be found for consecutive MPS molecules rotated by a (±n ⋅ 60°) angle different from 0°, a helicoidal arrangement of the organic OSDAs should be developed. Combined with the paired incorporation of MPS⋅⋅⋅Mg through the development of H‐bonds, this would induce a helicoidal distribution of Mg dopants embedded in the achiral AFI channels, arising a new type of chirality in zeolite materials that could potentially lead to asymmetric catalysts.^[25]^ For this reason, we calculated the stability for the different rotation angles between consecutive MPS cations, keeping always the Mg dopant in the position driven by the H‐bonds developed with MPS, as determined by the structure‐solution study. MPS cations were loaded in the position found by Rietveld, and manually rotated to the required angle, building systems with complete helical pitches. The relative stability was then calculated by DFT geometry‐optimization of the systems. Unfortunately, relative energy results (Table S6 in the Supporting Information) suggested that the most stable rotation between MPS cations corresponded indeed to 0°, which is the only angle that would not lead to chiral distributions of dopants. In fact, this must correspond to the actual case in MgAPO‐5/MPS since, should a helicoidal arrangement of Mg⋅⋅⋅MPS occur, this would lead to a space group with an helicoidal symmetry axis, what was not observed in the 3DED study.

## Discussion

Our work shows that MPS cations are able to drive the incorporation of Mg dopants through the development of H‐bonds between the hydroxyl groups and the O(Mg) framework atoms. Interestingly, we found that this is not particular for Mg dopants, but the same occurs for Zn or Si dopants, as evidenced by the presence of the same extra‐diffractions in these materials as those found in MgAPO‐5 (see Figure S14 in the Supporting Information). Hence, MPS can drive the incorporation of any low‐valent dopant in AFI materials, with divalent dopants replacing Al^3+^ and also Si^4+^ replacing P^5+^, since both involve generation of a negative charge.

Worth is noting that in our AFI/MPS model there is a preferred orientation of the MPS cations along the polarized 12‐MR AFI channels, with phenyl rings pointing always towards −c, involving that crystal growth of the AFI channels, which occurs in the direction of polarization,[^40^, ^41^] only occurs by adding MPS cations in a particular orientation, incorporating first the charged ammonium‐containing side of MPS upon interactions through H‐bonds with Mg units, and then the hydrophobic aromatic side is dragged.

When we analyzed the structure‐directing effect of the diastereoisomer (1*R*,2*S*)‐N‐methyl‐ephedrine (MEP) (see Figure S15 in the Supporting Information), we observed that the special symmetry associated to the particular incorporation of MPS occurred to a much lower extent in this case, being only partially apparent in the sample obtained at 140 °C (Figure S15‐A). Fluorescence results (Figure S15‐C) showed a stronger incorporation of this MEP isomer as dimers, what explains the lower ability of this cation to promote an ordered distribution of dopants that is associated to the incorporation as monomers. Indeed, ^13^C NMR (Figure S15‐D,E) showed much broader signals compared to those of MPS cations (Figure 1), evidencing a less ordered MEP incorporation. On the other hand, the dimethyl quaternary ammonium derivatives (1*S*,2*S*)‐N,N‐dimethyl‐pseudoephedrinium and (1*R*,2*S*)‐N,N‐dimethyl‐ephedrinium, even despite being incorporated as monomers,^[42]^ however they do not promote this type of special arrangement (Figure S16); this might be associated to the lack of a conformational rigidity as that of MPS imposed by the presence of strong intramolecular N−H⋅⋅⋅OH H‐bonds, which is not present in these quaternary ammonium cations since they lack N−H bonds, resulting in a wide conformational freedom. Finally, the removal of a methyl group from MPS, giving the original pseudoephedrine, also involves a notable reduction of the ability to arrange in this type of configuration since the associated extra XRD peaks are scarcely observed, only in samples obtained with high water and low organic and Mg contents (we had seen that this reduces the supramolecular aggregation), what is probably associated to the higher trend of pseudoephedrine to arrange as dimers.^[29]^

In sum, our study shows that MPS cations have the right molecular structure that enables to have a rigid intramolecular H‐bonded conformation that promotes a control over the incorporation of low‐valent dopants through the development of H‐bonds with hydroxyl groups. Unfortunately, there is only one type of crystallographic position in the AFI framework, and consequently no special selectivity during a catalytic reaction would be expected for this material. However, this strategy of using hydroxyl‐containing OSDAs opens the way for driving the Al incorporation in other complex zeolitic frameworks with several crystallographic positions like MFI, BEA or FER, among others, especially considering that the presence of Al should increase the hydrophilicity of the zeolite. Work is currently under way in our group towards this aim.

## Conclusions

(1*S*,2*S*)‐N‐methyl‐pseudoephedrine displays a particular molecular structure that enables to drive the spatial incorporation of divalent dopants embedded in the AFI aluminophosphate network through the development of H‐bonds with the negatively‐charged O atoms surrounding the dopant, leading to an ordered distribution of the active sites in the microporous network. Such spatially‐ordered incorporation of OSDA⋅⋅⋅dopant pairs is a direct consequence of the molecular structure of the OSDA, which contains intramolecular H‐bond forming groups that imposes a conformational rigidity to the molecular structure, while allowing to establish strong H‐bonds with the dopant TO_4_ units. Thus, this work provides a crucial step towards the long‐time sought objective of controlling the incorporation of active sites in catalytic zeolite materials by introducing a new strategy given by the use of hydroxyl‐containing OSDAs able to develop H‐bonds with low‐valent dopants.

## Experimental Section

### Synthesis of (1S,2S)‐N‐methyl‐pseudoephedrine

(1S,2S)‐N‐methyl‐pseudoephedrine (MPS) was prepared from (1S,2S)‐pseudoephedrine following the Leucart reaction. Details are given in the Supporting Information.

### Synthesis of MPS‐MgAPO‐5

Nanoporous Mg‐doped aluminophosphate materials were prepared by hydrothermal methods using MPS as OSDA. Unless otherwise stated, the molar composition of the synthesis gels was 1MPS : 1P_2_O_5_ : 0.16MgO : 0.92Al_2_O_3_ : 50H_2_O. In a typical preparation, the corresponding amounts of H_2_O, H_3_PO_4_ (Sigma‐Aldrich, 85 % in water), pseudoboehmite (Pural SB‐1 77.5 % Al_2_O_3_, Sasol) and Mg(CH_3_COO)_2_ ⋅ 4H_2_O (Sigma‐Aldrich, 99.5 %) were stirred for 1 h, after which MPS was added and the mixture was stirred for additional 2 h, giving a pH around 4. The gels were introduced into 60 ml Teflon lined stainless steel autoclaves and heated statically at 140 or 180 °C under autogenous pressure for 24 h. The resulting solids were separated by filtration, washed with ethanol and water and dried at room temperature overnight. Details of the general physico‐chemical characterization of the materials are given in the Supporting Information.

### 3D‐electron diffraction studies and Rietveld refinement

The three‐dimensional electron diffraction (3DED) data were collected with continuous rotation electron diffraction (cRED) technique^[43]^ using a 200 kV JEOL JEM‐2100 transmission electron microscope equipped with quad hybrid pixel detector (Timepix, 512×512 pixels with the size of 55 μm, Amsterdam Sci. Ins.). The 3DED Data were processed using X‐ray Detector Software (XDS),^[44]^ which generated the integrated intensities used for structure solution and refinement. The framework structure was solved using the software SHELXT.^[45]^ The 3D reciprocal lattice was reconstructed using REDp software.^[46]^ The synchrotron powder X‐ray diffraction used for Rietveld refinement was collected at beamline BL04‐MSPD in ALBA, the Spanish synchrotron radiation facility, under ambient temperature in Debye‐Scherrer mode with a wavelength of 0.61942 Å over a range of 2° to 40° with a step length of 0.001°. The Rietveld refinement was performed using Topas 6.0.^[47]^ The method of location of the OSDA position was as described before.^[48]^

### Racemization study

As‐made MgAPO‐5 solids with occluded MPS molecules were dissolved in 5 M NaOH aqueous solution, which were stirred for two hours at room temperature until complete dissolution of the solids. The released organic material was then extracted from the aqueous solution with diethyl‐ether in a separatory funnel. The organic phase was dried with K_2_CO_3_, and the solvent was roto‐evaporated, leading to an oily product. ^1^H and ^13^C NMR demonstrated this to be pure MPS. Retention of the enantiomeric purity was then studied by polarimetry, using a P1000 LED A polarimeter (Krüs‐Optronic).

### Computational Details

In order to analyze the effect of MPS over the dopant location within the AFI network, molecular simulations based on a combination of molecular mechanics and quantum mechanics (DFT) were performed. We have already shown in previous works that the combined presence of rotatable bonds and H‐bond donor and acceptor groups, with the consequent possibility of developing intramolecular H‐bonds, drives the conformational space of these molecules, which can strongly affect their structure‐directing behavior;[^29^, ^32^] therefore, an initial conformational analysis was performed. The conformational space of MPS was scanned by means of the Conformers Calculation Module in Materials Studio,^[49]^ optimising the molecular structures for each set of dihedral angles, using the cvff force‐field;^[50]^ in these calculations, the atomic charge‐distributions of the (+1) protonated molecules were obtained from DFT+D calculations, using the B3LYP hybrid functional and the ESP charge calculation method, setting the total net charge to +1. Calculations of the stability of these conformers in vacuum were then performed at ab‐initio level with the Gaussian09 code,^[51]^ using DFT and the hybrid B3LYP functional (including the D3 Grimme dispersion term); a 6–311G++(d,p) basis set was employed. This initial study clearly showed a particularly stable conformation for this molecule. Calculation of the NMR chemical shielding of the different conformers (in vacuo) were performed with the gauge‐including projector augmented‐wave method (GIPAW) developed by Pickard and Mauri.^[52]^ The chemical shift for a nucleus in a given position (δ(r)) is defined as:

$$\delta \left(r\right)=\sigma {}_{\mathrm{ref}}-\sigma \left(r\right)$$

where σ(r) is the isotropic shielding obtained in the calculations. In order to compare with the experimental ^13^C chemical shifts, we chose a σ_ref_ of 180 ppm, so that the theoretical and experimental chemical shifts of the most shielded nuclei roughly coincide.

The location and stability of MPS in the Mg‐containing AFI framework was then studied by DFT calculations. The initial location of the molecules and the AFI unit cell was that obtained after the Rietveld refinement. Depending on the rotation angle between consecutive MPS cations, different AFI supercells were used in order to match and complete one helical pitch (1×1×1 for 0°, 1×1×2 for 180°, 1×1×3 for 120 and −120°, and 1×1×6 for 60° or −60°). These systems were geometry‐optimized at DFT+D level, which were performed using plane‐waves as basis set (with a cut‐off of 489.8 eV), and the PBE generalized gradient approximation as functional^[53]^ with the Grimme dispersion term, as implemented in the CASTEP module^[54]^ in Material Studio. Relative energies were obtained by subtracting the energy of the most stable system; all energies are reported in kcal/mol per AFI unit cell.

## Conflict of interest

The authors declare no conflict of interest.

1

## Supporting information

As a service to our authors and readers, this journal provides supporting information supplied by the authors. Such materials are peer reviewed and may be re‐organized for online delivery, but are not copy‐edited or typeset. Technical support issues arising from supporting information (other than missing files) should be addressed to the authors.

Supporting Information

## Data Availability

The data that support the findings of this study are available in the supplementary material of this article.
