# Supplementary material for: Driving the Active Site Incorporation in Zeolitic Materials via the Organic Structure‐Directing Agent Through Development of H‐Bonds with Hydroxyl Groups
Source: Chemistry. 2022 Jun 8;28(42):e202200702. doi: 10.1002/chem.202200702 (PMC9400953; doi:10.1002/chem.202200702)
Supplement: Supplementary file 1 — Supporting Information [file CHEM-28-0-s001.pdf]

Chemistry–A European Journal

Supporting Information

Driving the Active Site Incorporation in Zeolitic Materials via the Organic Structure-Directing Agent Through Development of H-Bonds with Hydroxyl Groups

Beatriz Bernardo-Maestro, Jian Li, Joaquín Pérez-Pariente, Fernando López-Arbeloa, and Luis Gómez-Hortigüela*

Synthesis of (1*S*,2*S*)-N-methyl-pseudoephedrine

25.0 g of (1*S*,2*S*)-pseudoephedrine (Sigma-Aldrich, 98 %) were carefully added (with cooling) to a solution of 15.1 g of formaldehyde (Sigma-Aldrich, 37 wt % aqueous solution) and 23.0 g of formic acid (Sigma-Aldrich, 95%), and the mixture was refluxed overnight. 18.2 g of 37 wt % HCl (Panreac) were then carefully added, and this aqueous phase was washed in a separatory funnel with diethyl ether. The aqueous phase was collected, and a solution of NaOH (25 wt%, around 160 mL) was added until reaching a pH of 12, after which an oily product was formed. This was then extracted with diethyl ether (3x100 mL), the organic phase was finally dried with K_2CO_3, and the solvent was evaporated. (1S,2S)-N-Methyl-pseudoephedrine was obtained with ~80 % yield. ^{1}H NMR ($CDCl_3$): 0.74 (d, 3H, C**H**$_3$C), 2.33 (s, 6H, C**H**$_3$N), 2.59 (dq, 1H, C**H**N), 4.22 (d, 1H, C**H**OH), 5.11 (broad band, 1H, OH), 7.26-7.41 (m, 5H, aromatics). ^{13}C NMR ($CDCl_3$): 7.30 (**C**H$_3$C), 40.9 (**C**H$_3$N), 66.9 (C**H**N), 75.9 (**C**HOH), 128.4-129.2 (unsubstituted aromatic C), 143.1 (substituted aromatic C). Retention of the enantiomeric purity was confirmed by polarimetry (α(1S,2S-MPS) = +45º; α(1R,2R-MPS) = –45º; c = 5 g/100mL in methanol).

General physico-chemical characterization details:

The obtained solids were characterized by powder X-Ray Diffraction (XRD), using a Philips X´PERT diffractometer with CuK_α radiation with a Ni filter. Thermogravimetric analyses (TGA) were registered using a Perkin-Elmer TGA7 instrument (heating rate = 20ºC/min) under air flow. The crystal morphology of the materials was studied by scanning electron microscopy (SEM) using a Hitachi TM-1000 Tabletop microscope. The aggregation state of the molecules in the solid samples was studied by UV-VIS fluorescence spectroscopy, using a RF-5300 Shimadzu fluorimeter in front-face configuration.

Solid State MAS NMR details:

Solid State MAS-NMR spectra of the solid samples were recorded with a Bruker AV 400 WB spectrometer, using a BL7 probe for ^{13}C and a BL4 probe for ^{31}P. ^{1}H to ^{13}C Cross-Polarization spectra were recorded using π/2 rad pulses of 4.5 ms for ^{1}H, a contact time of 5 ms and a recycle delay of 3 s. For the acquisition of the ^{13}C spectra, the samples were span at the magic angle (MAS) at a rate of 5-5.5 kHz. For ^{31}P, π/2 rad pulses of 4.25 ms and recycle delays of 80 s were used; these spectra were recorded while spinning the samples at *ca.* 10 kHz.

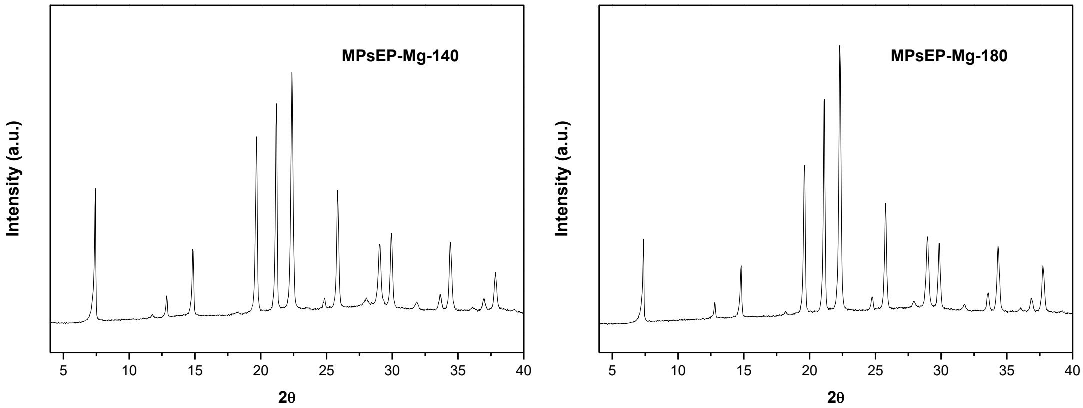

Figure S1. XRD patterns of MgAPO-5/MPS at 140º (left) or 180º (right) crystallization temperatures.

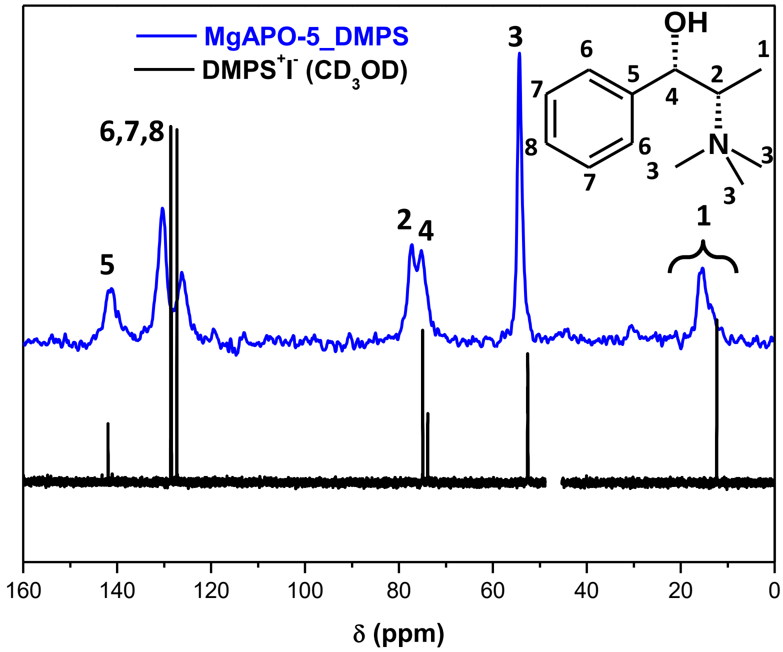

Figure S2. ^{13}C CP MAS NMR spectra of MgAPO-5 materials obtained with DMPS (blue) at 140 ºC. Liquid ^{13}C NMR of the iodide salt in CD_3OD is shown in black lines for comparison.

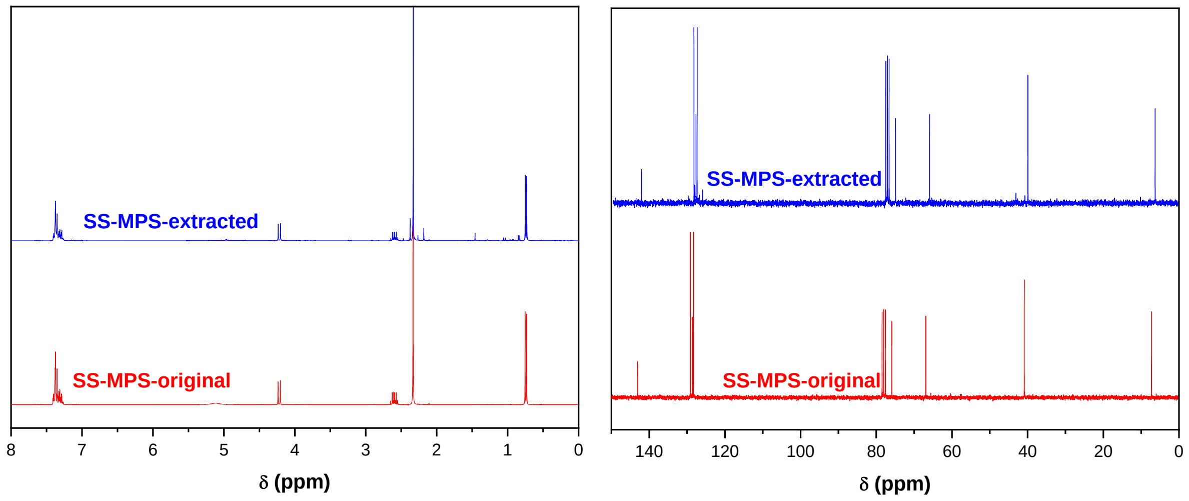

Figure S3. ^{1}H (left) and ^{13}C (right) NMR of (1S,2S)-N-methyl-pseudoephedrine (in $CDCl_3$) after release from MgAPO-5/MPS solids (blue); original spectra are shown in red for comparison.

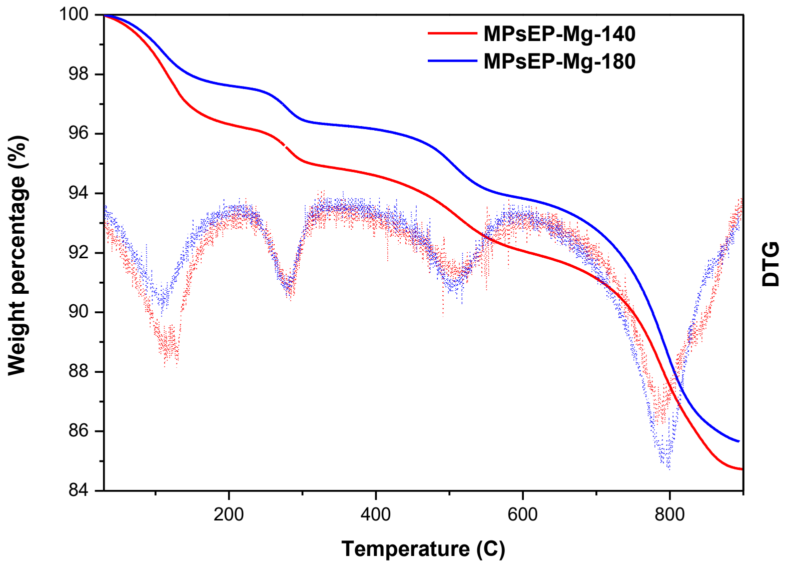

Figure S4. TGA (solid lines) and derivative (dashed line) of MgAPO-5/MPS at 140º (red) or 180º (blue) crystallization temperatures.

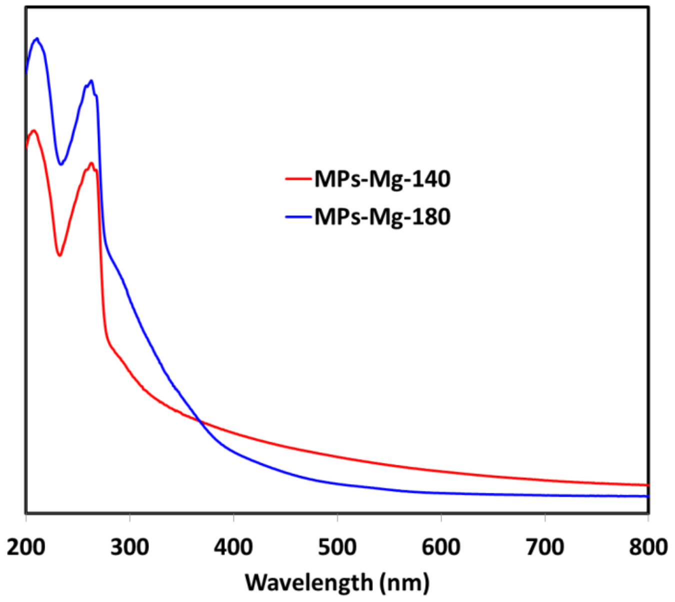

Figure S5. UV-Vis Diffuse reflectance spectra of MgAPO-5/MPs at 140º (red) or 180º (blue) crystallization temperatures.

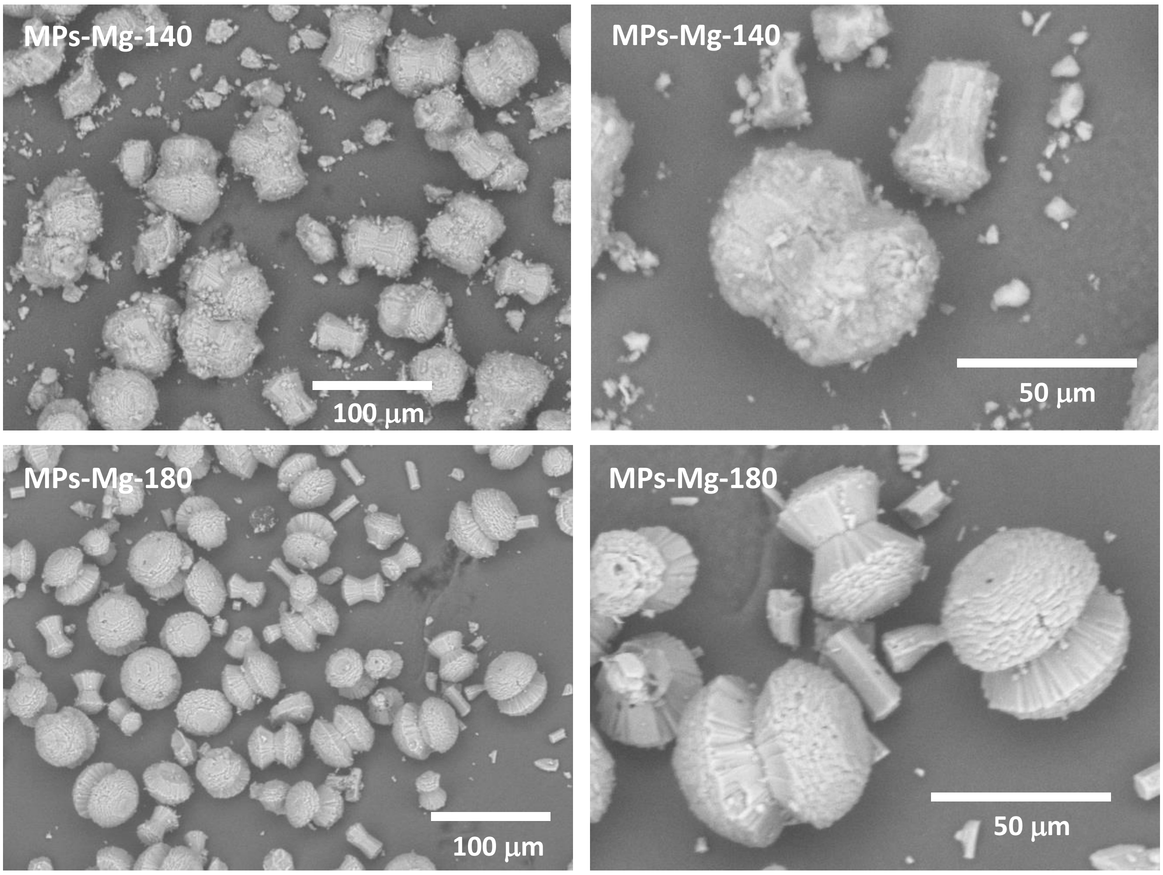

Figure S6. SEM (at two magnifications, left and right) of MgAPO-5/MPs at 140º (top) or 180º (bottom) crystallization temperatures.

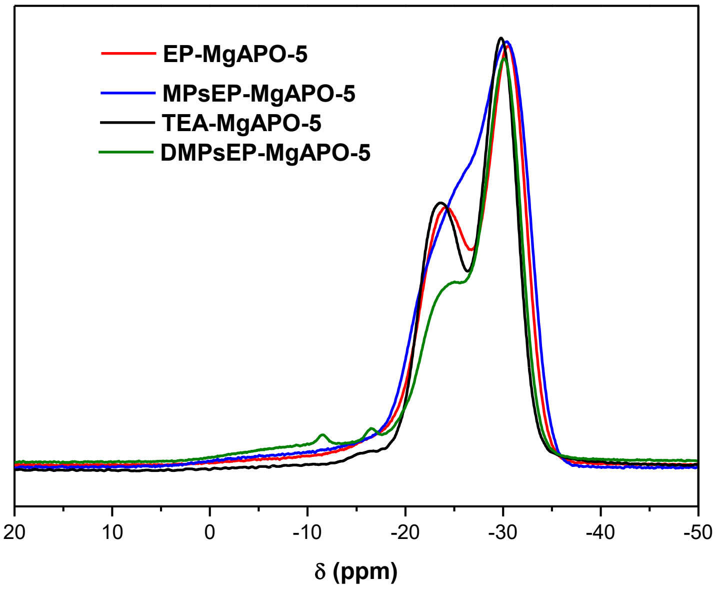

Figure S7. Comparison of the ^{31}P MAS NMR spectra of several MgAPO-5 materials obtained with (1R,2S)-ephedrine (red), (1S,2S)-N-methyl-pseudoephedrine (blue), (1S,2S)-N,N-dimethyl-pseudoephedrinium (green) or triethylamine (black) as SDA.

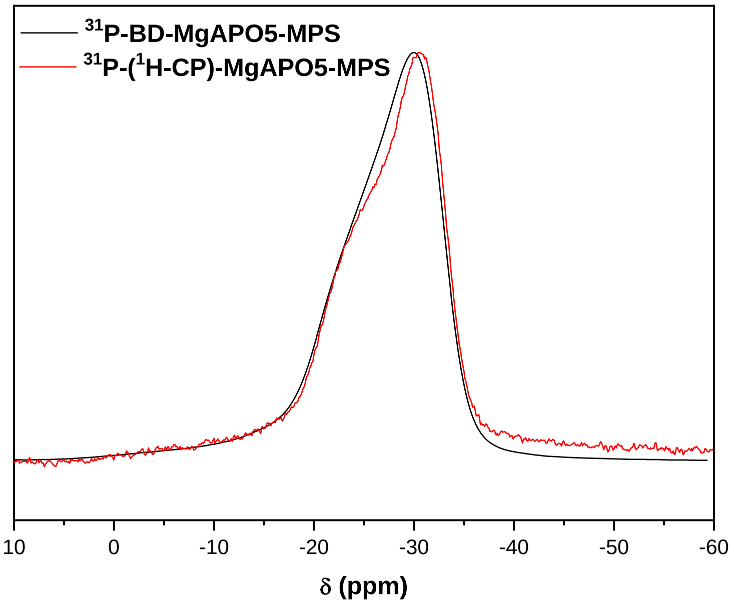

Figure S8. Comparison of the ^{31}P BD (black line) and CP (red line) MAS NMR spectra of MgAPO-5/MPS materials obtained at 180 ºC.

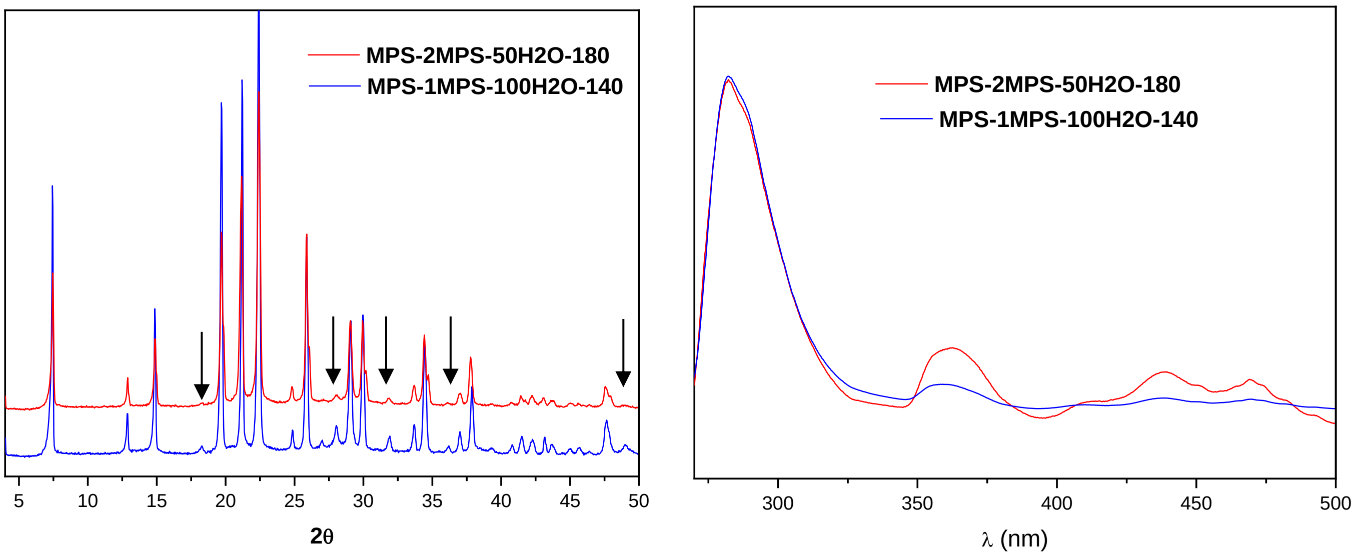

Figure S9. XRD patterns (left) and height-normalized fluorescence spectra (right) of MgAPO-5/MPs solids obtained with low MPS concentration and at low crystallization temperature (blue) or high MPS concentration and high crystallization temperature (red), where the latter shows a higher occurrence of aggregates and a lower intensity of the extra-XRD peaks (indicated by arrows).

Table S1. Relative energies (in kcal/mol) of the different conformers, calculated at different levels of theory.

Conformer	DFT/B3LYP (Gaussian)	DFT/PBE (CASTEP)	cvff (Forcite)
**I**	0.0	0.0	0.0
**II**	3.2	3.9	3.0
**III**	3.7	3.9	5.2
**IV**	5.9	5.7	6.7
**V**	9.3	9.1	11.5
**VI**	6.6	6.0	11.6
**VII**	7.1	6.6	12.9

Table S2. Calculated (at DFT/B3LYP level) ^{13}C NMR chemical shifts (in ppm) of protonated MPs (in vacuo) in the two stable conformations (labelling as in Figure 1), and comparison with experimental ^{13}C NMR of MPs hydrochloride (in aqueous solution).

Conformer	1	4		2	3	6		7		8	5
I	7.9	36.5	43.5	77.8	79.4	128.6	134.5	137.5	137.1	140.0	138.5
II	18.8	42.3	48.4	77.8	79.2	123.3	130.3	136.8	138.6	138.7	143.1
Exp	7.5	35.2	42.3	66.1	72.6	127.3		129.08			139.1

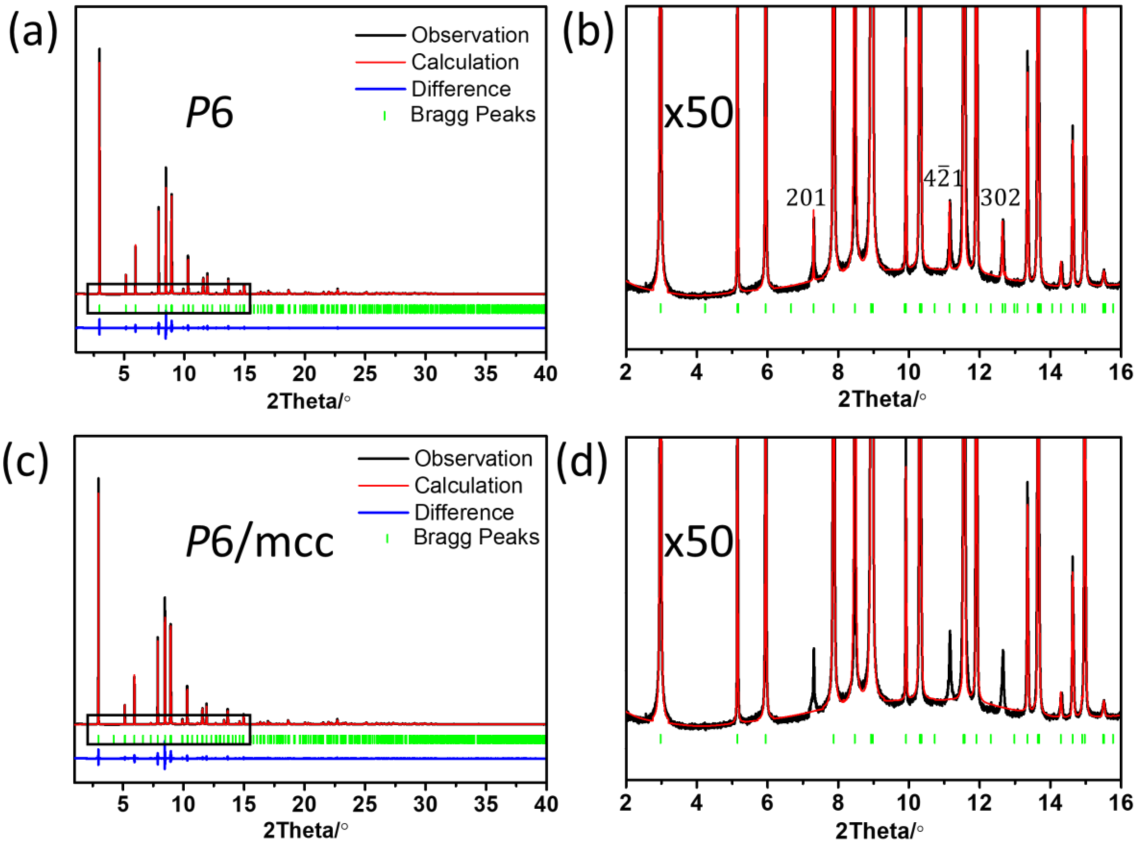

Figure S10. Pawley profile-fitting plot of MgAPO-5/MPS with space group of *P*6 (a, b) and *P*6/*mcc* (c, d). (b) and (d) are the 50-times scaled up of (a) and (c) at the 2θ angle of 2~16°, respectively. Black, red and blue curves are for experimental, calculated, and difference values, respectively; vertical green lines are for allowed reflections of MgAPO-5/MPS.

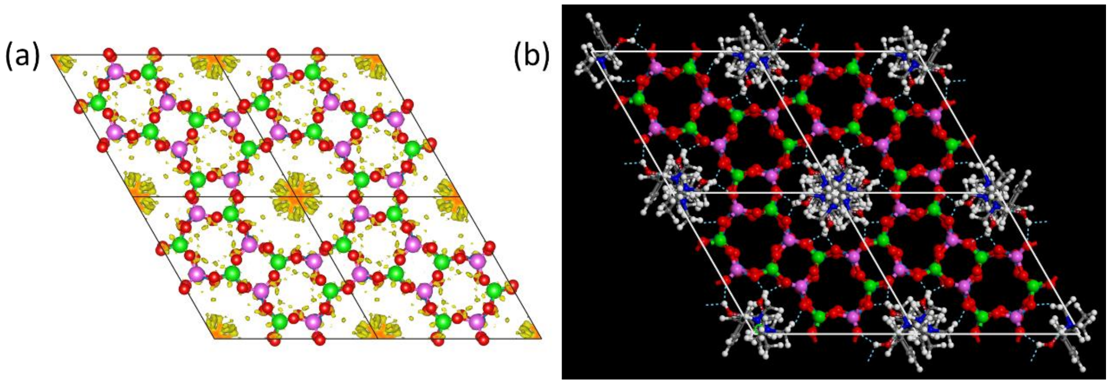

Figure S11. Difference electron density maps of MgAPO-5/MPS that views along [001] (a), after initial scaling, showing the positive only. (b) Structure model views along [001] (a) with disordered OSDA and doped Mg.

Table S3. cRED: Experimental parameters, crystallographic data and structure refinement details of MgAPO-5/MPS.

**Experimental parameters and crystallographic data**	
Number of datasets	4
Tilt step	0.23°
Wavelength	0.0251 Å
Program for data procession	*XDS*
Program for structure solution	ShelxT
Crystal system	Hexagonal
Unit cell dimensions	*a* = 13.7916(2)Å<br>*c* = 8.3886(7)Å
Volume	1381.81(12)$Å^3$
Possible space group	*P6*, *P-6*, *P622*, *P6mm*, *P-62m*, *P6/mmm*
Resolution	0.79 Å
Completeness	100%
R_{int}	32.10%
No. of reflections	17581
No. of unique reflections	1838
**Structure Refinement against cRED data**	
Formula	$Mg_1Al_{11}P_{12}O_{48}$
Crystal system	Hexagonal
Space group	*P6*
Unit cell dimensions	*a* = 13.7916(2)Å<br>*c* = 8.3886(7)Å
Volume	1381.81(12)$Å^3$
Dataset (*h*,*k l*)	-16~16, -14~17, -9~10
Tot., Uniq. Data, Rint	17581, 1838, 31.49%
Observed Data [$I > 2.0\sigma(I)$]	980
$N_{reflections}$, $N_{parameters}$, $N_{restraints}$	1838, 49, 1
R_1, wR_2, *Gof*	0.1907, 0.4513,1.310
I/σ	6.1
ρ_{min}, ρ_{max} (e^- /$Å^3$)	-0.4/0.2

Table S4. Rietveld Refinement of MgAPO-5/MPS.

Empirical formula	$Al_{11}MgP_{12}O_{48}(C_{11}NOH_{18})$
Wavelength	0.61942 Å
Radiation	Synchrotron Radiation
Crystal system	Hexagonal
Space group	*P6*
Unit cell dimensions	a = 13.78995(13)Å<br>c = 8.38816(7)Å
Volume	1381.41(3)$Å^3$
Z	1
2ϑ range for data refinement	$2° < 2\vartheta < 40°$
Number of parameters	79
Number of reflections	708
Number of data points	12004
Number of restrains	8 for Al-O, 8 for P-O and 32 for O-T-O and T-O-T
Refinement method	Rietveld refinement
R_p/R_{wp}	0.1014/0.1349

Table S5. Al-O and P-O bond distances (Å) and Bond angels (°) for MgAPO-5/MPS.

.

Bond angles (°)	Min	Max
T-O-T	138.1(3)	168.0(1)
O-T-O	103.4(1)	116.2(1)
**Bond distances (Å)**	**Min**	**Max**
Al-O	1.66(4)	1.78(4)
P-O	1.48(4)	1.54(4)

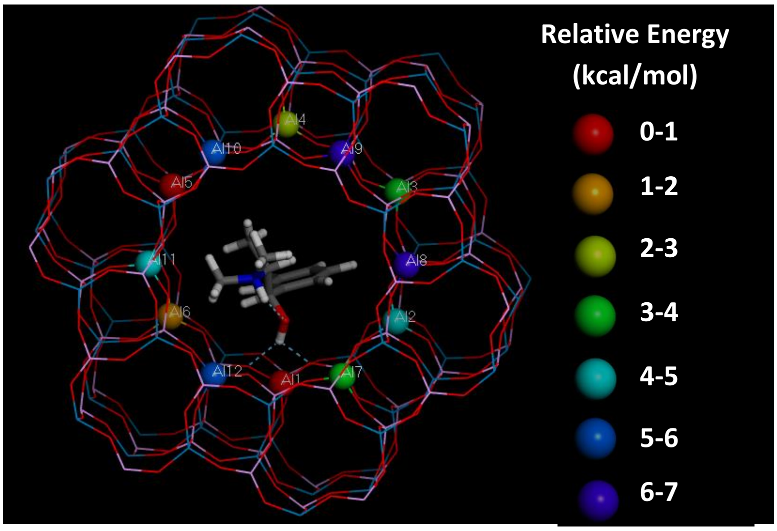

Figure S12. Labelling for the different Mg positions with respect to MPS, and DFT calculated relative energies with the corresponding color code.

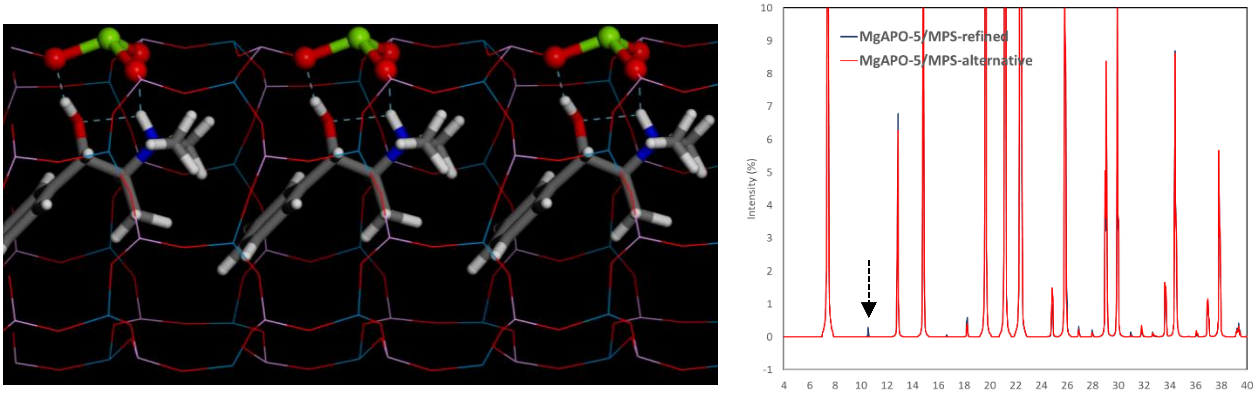

Figure S13. Alternative location for MPS cations with double H-bond interaction (left), and theoretical XRD pattern (blue line) compared with the one for the Rietveld model (red line). An arrow highlights the additional peak that should appear with the alternative location.

Table S6. Relative energies (in kcal/mol u.c.) of MPs cations arranged as supramolecular helices of different angles, with Mg following the helicoidal pattern.

Angle	Symmetry	R.E. (-c)
**-120**	$P3_2$	12.2
**-60**	$P6_5$	7.3
**0**	P1	0.0
**60**	$P6_1$	6.1
**120**	$P3_1$	16.8
**180**	$P2_1$	3.7

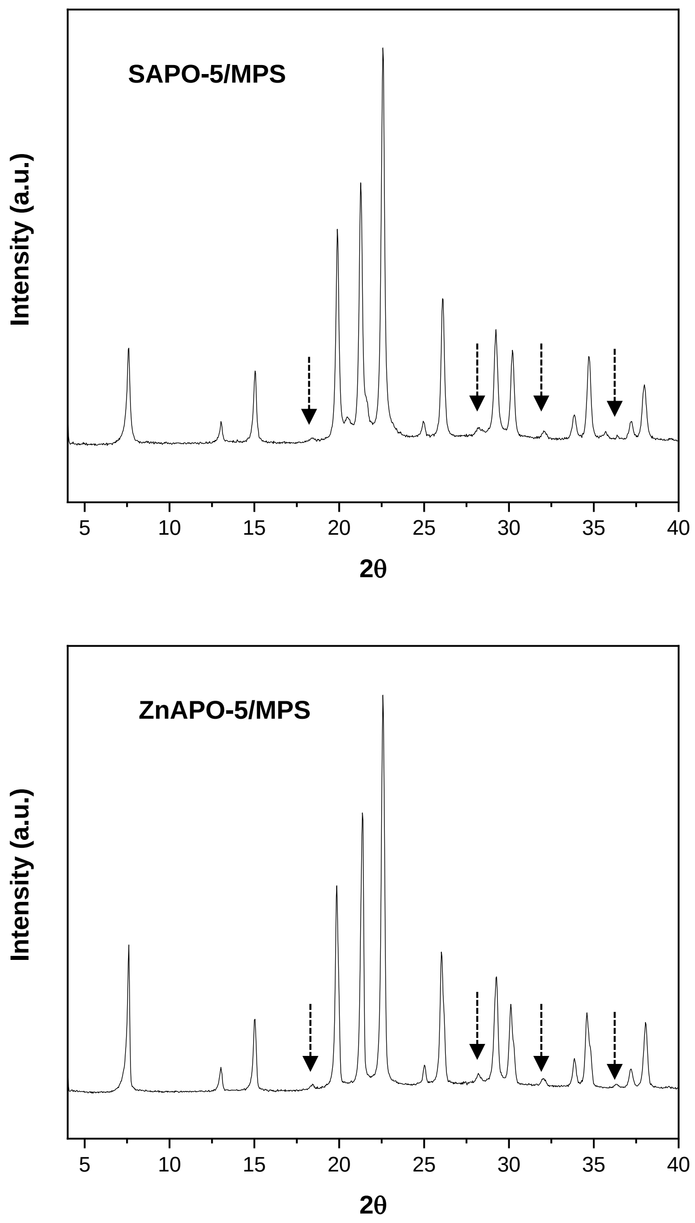

Figure S14. XRD patterns of SAPO-5 (top) and ZnAPO-5 (bottom) materials obtained with MPS; arrows indicate the additional diffraction peaks corresponding to the special *P*6 symmetry.

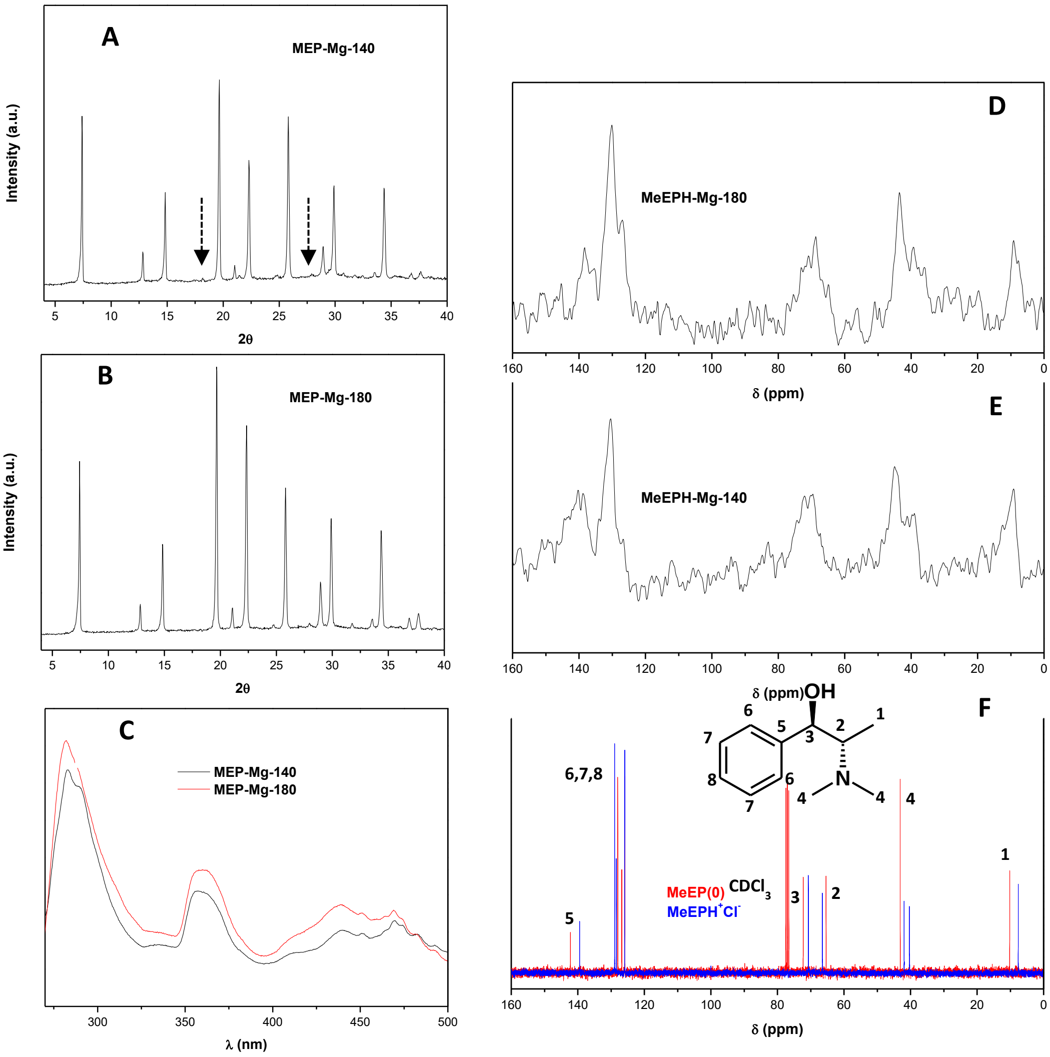

Figure S15. Physico-chemical characterization of MgAPO-5 materials obtained with (1R,2S)-N-methyl-ephedrine (MEP). A and B: XRD patterns obtained after crystallization at 140º (A) or 180º (B) C: UV-VIS fluorescence spectroscopy. D and E: ^{13}C CP MAS NMR. F: ^{13}C NMR of solutions of neutral MEP (red) and the protonated salt (blue).

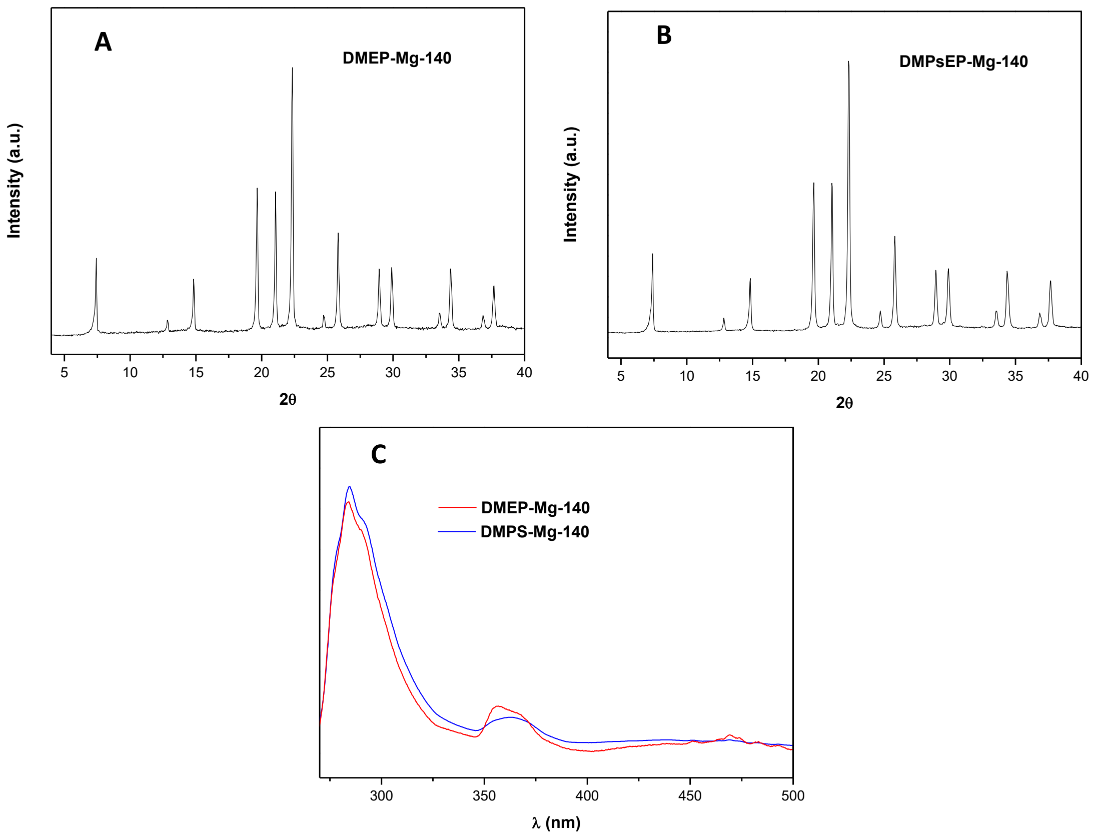

Figure S16. Physico-chemical characterization of MgAPO-5 materials obtained with (1R,2S)-N,N-dimethyl-ephedrinium (DMEP) or (1S,2S)-N,N-dimethyl-pseudoephedrinium (DMPS). A and B: XRD patterns obtained after crystallization at 140º (A). C: UV-VIS fluorescence spectroscopy.
